# Cutaneous Leishmaniasis Mimicking a Nasal Tumor: A Case Report

**DOI:** 10.1155/crdi/5535402

**Published:** 2025-11-10

**Authors:** Maryam Hekmat, Mohammad Reza Namazi, Amir Hossein Najibi, Babak Shirazi Yeganeh, Negin Fazelzadeh Haghighi

**Affiliations:** ^1^Molecular Dermatology Research Center, Shiraz University of Medical Sciences, Shiraz, Iran; ^2^Department of Dermatology, Shiraz University of Medical Sciences, Shiraz, Iran; ^3^Student Research Committee, Fasa University of Medical Sciences, Fasa, Iran; ^4^Department of Pathology, Shiraz University of Medical Sciences, Shiraz, Iran

**Keywords:** leishmania, nose, tumor, ulcer

## Abstract

Leishmaniasis is a disease caused by *Leishmania* protozoa that is transmitted to the patient by sand flies. Depending on the *Leishmania* species, the disease can present with cutaneous, mucosal, or multiorgan involvement. Cutaneous leishmaniasis can present with diverse clinical manifestations mimicking other diseases. We present a 26-year-old pregnant woman with a painless tumoral lesion on her nose. Due to the atypical clinical presentation of our case for leishmaniasis, it is worth reporting.

## 1. Introduction

Leishmaniasis is a disease caused by *Leishmania* protozoa that is transmitted to the patient by sand flies. It is more prevalent in poorer countries, and according to a 2023 review paper by Knight et al., endemic locations include Asia, the Middle East, North Africa, East Africa, the Mediterranean, and South and Central America [1]. Leishmania parasites are classified into Old World and New World categories. The most common species in the Old-World group are *Leishmania major*, *Leishmania infantum,* and *Leishmania tropica*. The New-World group consists of *Leishmania amazonensis*, *Leishmania chagasi*, *Leishmania mexicana*, *Leishmania braziliensis*, *Leishmania guayanensis*, and *Leishmania naiffi*. *Leishmania* species can cause cutaneous, mucosal, or multiorgan involvement. Cutaneous leishmaniasis (CL) usually manifests with papular lesions that gradually develop into ulcerative plaques. However, the illness can present with a variety of clinical symptoms that can mimic other dermatological conditions. It should be noted that the lesions caused by Old-World species are usually self-limited, but New-World species can cause more severe cutaneous or mucocutaneous lesions [2, 3]. Leishmaniasis diagnostic procedures include direct histopathology or parasite culture, as well as indirect serology or molecular diagnostics. Direct testing is the highly specific gold standard test. Direct testing with parasite culture in Novy–MacNeal–Nicolle medium is difficult, prone to contamination, and requires expertise [2]. The histopathologic changes in leishmaniasis include a diffuse infiltration of macrophages, lymphocytes, and plasma cells; occasionally, granulomatous inflammation is observed. Leishmaniasis can be easily recognized when amastigotes (parasite-filled histiocytes with kitenoplasts) are present. However, in late stages of the disease, amastigotes may disappear, making identification more difficult [4]. As indirect serology tests, including indirect fluorescent antibody (IFA), enzyme-linked immunosorbent assay (ELISA), western blot, and lateral flow assay, have low sensitivity, recent guidelines do not recommend them as a diagnostic tool for leishmaniasis. However, researchers are still trying to find a more specific and sensitive serology test for the diagnosis of leishmaniasis. Rapid test, a membrane-based qualitative immunoassay using polyclonal antibodies against peroxidoxin of *Leishmania* amastigote, is one of the methods that have been developed with variable accuracy. The Leishmania intradermal test (LST), intradermal injection of *Leishmania* extract, has been used for many years with good sensitivity and specificity, but its use has declined due to the scarcity of a standardized antigen [2]. Molecular testing for leishmaniasis, such as polymerase chain reaction (PCR), is more sensitive and specific than prior procedures. However, it requires experienced laboratory expertise. To have a simple and rapid molecular diagnostic test in resource-limited laboratories, isothermal platforms such as loop-mediated isothermal reaction (LAMP) and recombinase polymerase amplification (RPA) have been applied with good sensitivity and specificity. Methods used for the determination of *Leishmania* species include PCR in combination with restriction fragment length polymorphism (RFLP), multilocus enzyme electrophoreses (MLEE) which is limited to certain laboratories, and the use of monoclonal antibodies [2, 5]. Nonselective treatments used for limited CL include cryotherapy, photodynamic therapy, and thermotherapy. It is worth considering ultraviolet radiation, a cell-mediated immune response suppressor, as a treatment option for limited CL, but further investigation is needed for confirmation. To avert the damage to adjacent tissue, specific treatments are recommended. Selective therapies include intralesional pentavalent antimony, topical paromomycin, topical pentamidine, topical liposomal amphotericin B, and synthesized nitric oxide–releasing chitosan nanoparticles (NONPs). It should be noted that further investigation is needed to confirm the formulation of topical NONPs used for the treatment of CL. Systemic treatment is needed for more severe cases, including multiple lesions, lesions of the face or hard-to-reach areas, and immunosuppression. Pentavalent antimony, amphotericin B, and miltefosine are systemic treatment options are used for leishmaniasis. Newer options include immunotherapy with interferons and vaccines that are under investigation [6]. This report details a 30-year-old pregnant woman who appeared at our clinic with a substantial, erythematous, tumor-like lesion on her nose.

## 2. Case Presentation

**Figure 1:**
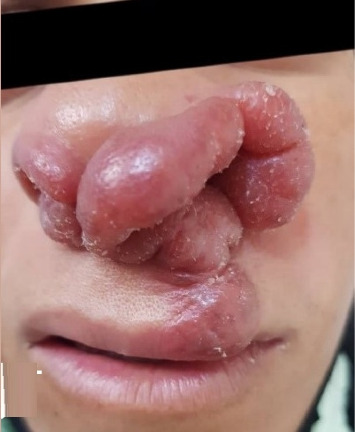
4 × 2.5-cm nonmobile, nonpulsatile tumoral lesion on the nose.

A 26-year-old pregnant woman was referred to the Dermatology Clinic of Shiraz University of Medical Sciences with a painless erythematous, 4 × 2.5-cm tumoral lesion on her nose that had been present for 2 years (Figure 1). The lesion was nonmobile, nonpulsatile, and not tender on palpation, and there was no ulcer surrounding or on the lesion. The general physical exam was normal, and no palpable lymph nodes were detected on the neck and axilla. In addition, initial lab data were requested, and no abnormality was seen.

**Figure 2:**
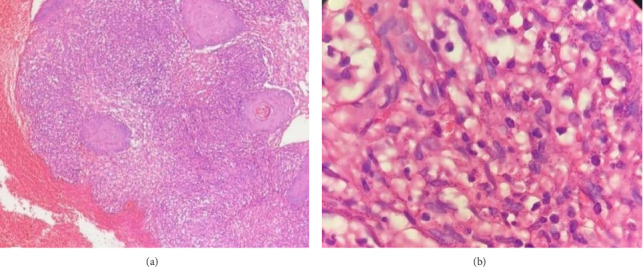
(a) Dermal infiltration of histiocytes, with admixture of plasma cells and lymphocytes. (b) Parasite-filled histiocytes (× 40 magnification).

A punch biopsy was performed. Our initial list of differential diagnosis included CL, cutaneous *tuberculosis* (lupus vulgaris), sarcoidosis, cutaneous B-cell lymphoma, Kaposi sarcoma (KS), and squamous cell carcinoma (SCC). Histopathological investigation indicated histiocyte infiltration of the dermis, along with plasma cells and lymphocytes. Many histiocytes contained tiny, uniform, spherical creatures, which a pathologist determined to be *Leishmania* amastigotes (Figures 2(a), 2(b)). Unfortunately, we did not have the laboratory techniques for determining the species of Leishmania. However, based on clinical presentation and prior studies [7] on the dominant species in Shiraz, Iran, the most likely species is *L. tropica*.

**Figure 3:**
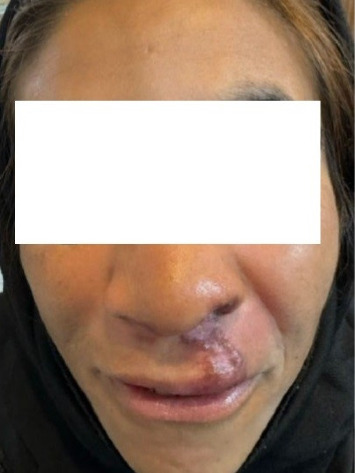
Complete resolution of the lesion after treatment, with only the surgical scar remaining.

Intravenous Amphotericin B was administered at a daily dose of 3 mg/kg as first-line treatment, but no significant response was observed after 7 days of treatment. After delivery, we administered 20 mg/kg/day intramuscular glucantime for 3 weeks, and due to the site and the large size of the lesion, surgical excision was performed. As shown in Figure 3, the lesion was completely resolved after surgery, with only the surgical scar remaining. The patient was seen at 6 months and 1 year after treatment, and no recurrence occurred.

## 3. Discussion

Cutaneous lesions of the nose occur in a variety of systemic and local diseases [8]. Patients may present with a tumor-like lesion, such as a papule, nodule, macule, or poorly circumscribed mass. While basal cell carcinoma (BCC) is the most common malignant tumor arising from the nasal skin, melanoma, SCC, and KS are other malignant lesions that carry a higher risk of morbidity and mortality. Additionally, inflammatory conditions such as sarcoidosis and rosacea may also present with large cutaneous papules and nodules on the nose [8, 9]. In the specific case of our patient, the indolent course of the lesion, as well as its nonulcerating, erythematous appearance was inconsistent with a diagnosis of melanoma or SCC. It is also less likely for a single, noninvasive lesion in an immunocompetent, otherwise healthy individual to be caused by KS or other systemic etiologies. This notion was further supported by the absence of any abnormal lab values as well as the absence of systemic signs/symptoms. The atypical appearance of our patient's lesion, which lacked any induration or central ulceration, was another notable finding. This elaborates the importance of holding a high clinical suspicion in areas where CL is endemic, even if the site and gross morphology of the lesion are not typical for such a diagnosis.

Although involvement of the exposed sites, including the face and extremities, is very common for CL, there is a limited number of reports describing an isolated lesion on the nasal skin. Bandyopadhyay and Bose have reported a case of CL presenting with a rhinophyma-like lesion on her nose with multiple satellite lesions on the surrounding skin. The patient responded well to a 3-month course of miltefosine treatment [10]. Bari and Ejaz also reported a 73-year-old man with rhinophyma-like CL successfully treated with meglutine antimonite [11]. Ramesh et al. have also reported a case of post–kala-azar dermal leishmaniasis occurring on the nose [12]. There seems to be a paucity of evidence on the true prevalence of nasal CL, with a study from Muzaffarabad district of Azad Jammu and Kashmir reporting a 20.2% prevalence of CL isolated to the nasal skin [13]. Future research should focus on conducting multicenter studies with a larger sample size to further explore the morphological features and therapeutic response of nasal CL.

Due to the site of involvement, and the clinical presentation of our case that is not typical for CL, this case is worth reporting.

## 4. Conclusion

Considering that CL can present with diverse clinical manifestations mimicking other diseases, physicians should be aware of this disease, especially in endemic areas.

## Data Availability

All data are available within the main text.
